# Comments on Cui Q‐Q *et al*: “Hippocampal CD 39/ENTPD 1 promotes mouse depression‐like behavior …”

**DOI:** 10.15252/embr.202050737

**Published:** 2020-08-12

**Authors:** Herbert Zimmermann

**Affiliations:** ^1^ Institute of Cell Biology and Neuroscience Goethe University Frankfurt am Main Germany

**Keywords:** Neuroscience

## Abstract

Comment on Hippocampal CD 39/ENTPD 1 promotes mouse depression‐like behavior through hydrolyzing extracellular ATP by Cui *et al*

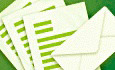

The paper of Cui *et al* (2020) published in EMBO *reports* requires further comment. First a short note on correct nomenclature: Protein and gene names are confused. CD39 (ectonucleoside triphosphate diphosphohydrolase 1, NTPDase‐1) is the protein name, whereas *ENTPD1* is the name for the corresponding (human) gene. In case of the mouse gene, this would be *Entpd1* (e.g., https://www.uniprot.org/uniprot/P49961 and https://www.genenames.org/about/guidelines/). The same applies to *ENPP1‐3* and *ENPP8*.

A central finding for the further development of the study of Cui *et al* concerns the increase in mRNA expression of *Entpd1* in hippocampal extracts of mice susceptible to chronic social defeat stress. In addition, the authors provide qPCR data to document gene expression of NTPDase‐2 and NTPDase‐3, two paralogs of NTPDase‐1, which—like NTPDase‐1—hydrolyze ATP to AMP. They observe a decrease in *Entpd2* mRNA, which is not further investigated but which is of relevance at least for the results concerning alterations in neurogenesis.

It is a major concern that the authors do not refer to relevant previous evidence indicating that the issue might be more complicated and might allow for alternative interpretations of their results. Cui *et al* conclude that CD39 affects hippocampal neurogenesis. Yet, they solely provide evidence for DCX‐positive neuroblast but not for mature neuron formation. They refer to the paper of Lin *et al* (2007), which shows high but non‐specified ectonucleotidase activity in the dentate gyrus. They do not mention previous papers which show that in the dentate gyrus, the ectonucleotidase NTPDase‐2 is specifically associated with hippocampal stem and progenitor cells (Shukla *et al*, 2005) and that deletion of NTPDase‐2 results in increased hippocampal progenitor cell and neuroblast proliferation (Gampe *et al*, 2015). Cui *et al* conclude that “Ectonucleotidase expressing in the SGZ of the hippocampus may serve as a brake on the proliferation of NSCs.” They do not mention an earlier paper on hippocampal SGZ (and subventricular zone) neurogenesis using mice globally null for *Entpd2* (Gampe *et al*, 2015), which concludes “This suggests that NTPDase2 functions as a brake on nucleotide‐mediated cell proliferation in either neurogenic niche.”

Cui *et al* introduce ARL67156 as “nonspecific inhibitor” of CD39. They do not mention that ARL67156 also inhibits NTPDase2 and NTPDase3 (Lévesque *et al*, 2007), particularly when the inhibitor is applied at high excess (injection of a 100 μM solution) over the endogenous extracellular ATP substrate. It should also be noted that injected apyrase not only mimics NTPDase‐1 but also NTPDase‐2 and NTPDase‐3.

Previous literature not mentioned in the paper of Cui *et al* could have helped to identify some of the hippocampal cellular elements involved. It has long been known that CD39/NTPDase‐1 is expressed by microglia and also by the vasculature of the brain (e.g., Braun *et al*, 2000; Färber *et al*, 2008). In fact, single‐cell RNA sequencing data (Zhang *et al*, 2014 and https://www.brainrnaseq.org/) show that NTPDase‐1 is expressed in microglia and endothelial cells but not in neurons or astrocytes. This contradicts Appendix Fig S1A–C of Cui *et al*, which implies that NTPDase‐1 immunoreactivity is expressed by essentially all hippocampal neurons. No microglia are discernible. A complete NTPDase1 Western blot lane revealing the specificity of the antibody would have been essential.

The picture of ectonucleotidase functions in chronic social defeat stress‐induced depression‐like behavior and hippocampal neurogenesis may therefore be more complex than inferred by Cui *et al* Microglial NTPDase‐1 may be mainly involved in the NTPDase‐1 effects observed. Regarding progenitor cell proliferation, progenitor cell‐associated NTPDase‐2 rather than NTPDase‐1 is likely to contribute. Whether ATP or the final hydrolysis product adenosine is more relevant remains to be investigated.
